# Under the Cartagena Protocol on Biosafety – Where is the Roadmap for Risk Assessment Taking Us?

**DOI:** 10.3389/fbioe.2015.00212

**Published:** 2016-01-21

**Authors:** Helmut Gaugitsch

**Affiliations:** ^1^Environment Agency Austria, Vienna, Austria

**Keywords:** Cartagena protocol, risk assessment and risk management, roadmap for risk assessment, biosafety, genetically modified organisms

## Abstract

The paper summarizes the history of the development of the guidance on risk assessment, including the roadmap under the Cartagena Protocol on Biosafety since 2008 until now. The aim and the contents of the roadmap for risk assessment of living modified organisms (LMOs) are described, in particular the five steps in the risk assessment process. After several rounds of discussions at the expert and political level, the guidance including the roadmap is currently revised taking into account the results of an in-depth practical testing process by the Parties, Non-Parties, and relevant organizations. The aim is to provide an improved version of the guidance for endorsement and broad support by the next meeting of the Parties to the Cartagena Protocol in December 2016.

## Brief History of the Roadmap Under the Cartagena Protocol on Biosafety

The Cartagena Protocol on Biosafety was adopted in January 2000 as a Protocol to the Convention on Biological Diversity (Secretariat of the Convention on Biological Diversity, 2000). It entered into force on 11 September 2003 after the ratification by 50 Parties. Currently (June 2015), it has been ratified by 170 Parties. The objective of the Protocol is to contribute to ensuring an adequate level of protection in the field of the safe transfer, handling, and use of living modified organisms (LMOs), specifically focusing on the transboundary movements. Due to consistency with the Convention on Biological Diversity, the term LMOs is used in the Protocol, according to the definition, this means the same as the term genetically modified organisms (GMOs) in other regulatory frameworks.

Risk assessment as the basis of decision making is at the heart of the Protocol, which contains in its Annex III further details on the objective, the use, the general principles as well as methodology and points to consider for an appropriate risk assessment.

Since the entry into force of the Protocol, Parties have discussed whether and how to complement the provisions of the Protocol on risk assessment by further guidance, providing more details to practitioners.

For that purpose, the Parties to the Protocol at their fourth meeting in May 2008 in Bonn, Germany, established an expert group of about 30 experts^1^ with the tasks to:

(a) develop a “Roadmap” on risk assessment of LMOs and
(b) explore the development of further guidance of risk assessment.

The expert group worked intensively to fulfill its tasks and provided recommendations to the various meetings of the Parties. The seventh meeting of the Parties in October 2014 in Pyeongchang, South Korea, decided a continuation of a so-called open-ended online forum,^2^ a continuation of the work of the expert group in an expanded form (six new members) and gave both – the online forum and the expert group – a new mandate (see chapters below).

## Contents of the Guidance on Risk Assessment of LMOs

The draft guidance on risk assessment of LMOs (Biosafety Clearing-House, 2012) in its current form consists of the following parts: Part 1 is the Roadmap for risk assessment of LMOs. Part 2 covers the risk assessment of specific types of LMOs and traits, with the following subchapters: living modified (LM) plants with stacked genes or traits, LM plants with tolerance to abiotic stress, LM trees, and LM mosquitoes. Part 3 covers the monitoring of LMOs released into the environment. Finally, there is a section on the use of terms (definitions).

This paper focuses on part 1 of the Guidance, the development of the roadmap for risk assessment of LMOs, in the following.

## The Contents of the Roadmap for Risk Assessment of LMOs

The roadmap is not meant to substitute the provisions of the Protocol, nor is it meant to be prescriptive or legally binding. It rather builds on and supplements Annex III of the Cartagena Protocol. It elaborates on how to undertake an LMO risk assessment, in particular by outlining the different steps in the risk assessment, suggesting several points to consider for each step and providing links to various sources of background material (peer reviewed literature, studies, and other publications by various institutions etc.). It is meant as a reference document for LMO risk assessors, those who conduct and/or review a risk assessment and it can serve as a training tool in capacity building.

It covers in principle all types of LMOs and all types of applications (field trials, placing on the market of commercial LMO products).

The main chapters are the following:

The chapter on “Overarching issues in the risk assessment process” describes which criteria the quality of scientific information should meet. It also describes which information including data may be considered relevant for the risk assessment. It outlines how uncertainty should be identified and considered throughout the risk assessment process.

The chapter on “Planning phase of the risk assessment” is crucial as it describes that the risk assessment should be embedded in a context, such as the existing policies, guidelines, and regulatory frameworks on biosafety in the respective country or region. Identification or clarification of protection goals, assessment endpoints, risk thresholds, and management strategies are important starting points for the risk assessment. In addition, this chapter refers to the process and criteria for the choice of the appropriate comparators and the challenges attached to that.

The chapter on “Conducting the risk assessment,” including the five steps is at the heart of the roadmap and is further described in the chapter below.

**Figure 1:**
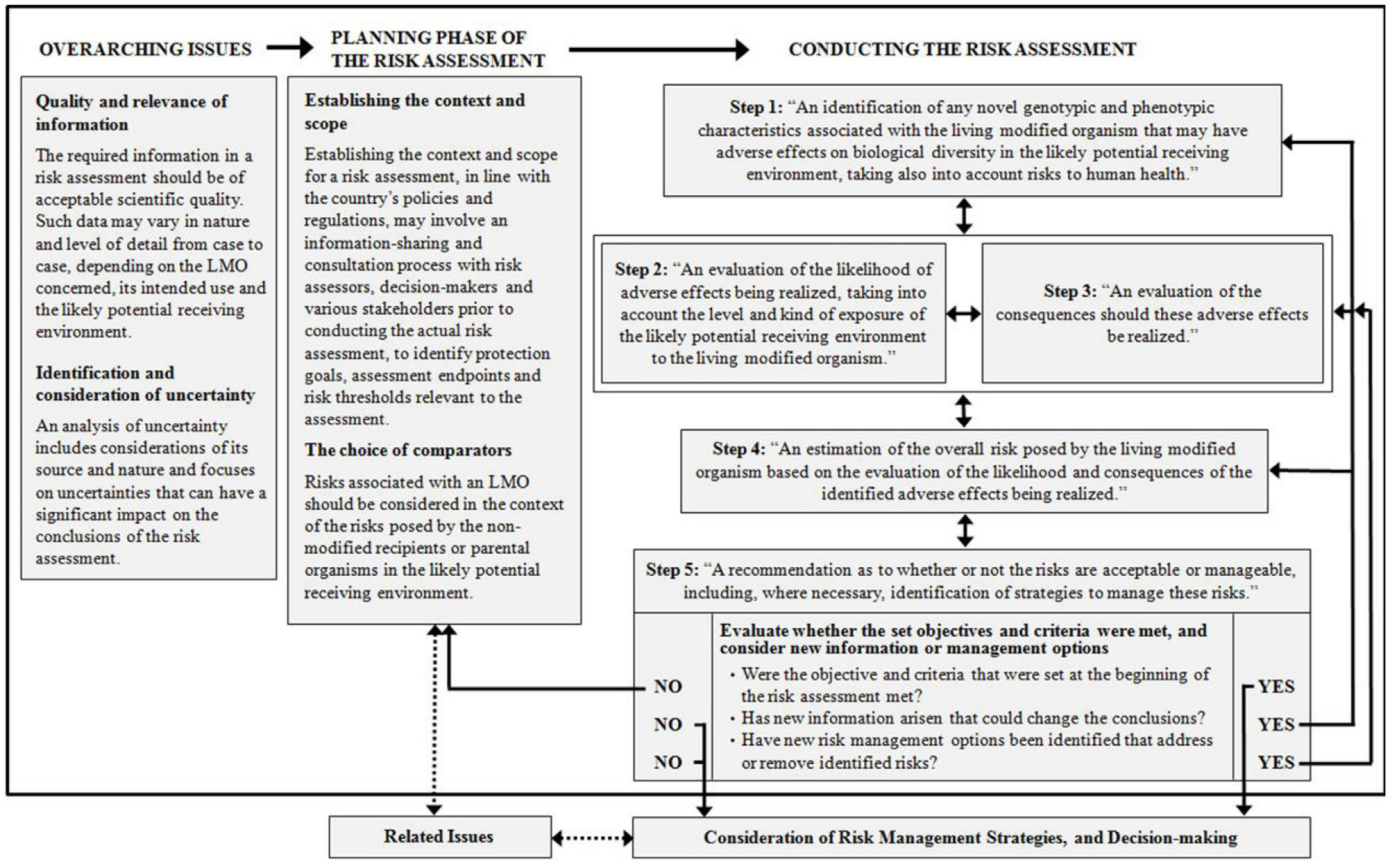
**Flowchart of the roadmap: (taken from Biosafety Clearing-House, 2012)**.

Figure 1 below refers to a Flowchart as a visualization of the roadmap.

## The Five Steps of the Risk Assessment Process

The roadmap in its core chapter outlines the following five steps in the risk assessment process:

Step 1: identification of the potential adverse effects of the LMO resulting from its genetic modification (“hazard identification”).
Step 2: evaluation of the likelihood of the adverse effect being realized.
Step 3: evaluation of the consequences should the adverse effect be realized.
Step 4: on the basis of Steps 1–3, the estimation of the risk, including the overall risk when all potential adverse effects, their likelihood and consequence are taken into account.
Step 5: recommendation on whether the risk is acceptable or manageable and any risk management strategies.

For each of the five steps, the roadmap establishes the rationale for the considerations in the respective step, any points to consider as well as provides links to relevant background material from various sources.

In Step 1, when identifying potential adverse effects, the rationale for the step is for example that the risk assessor needs to identify changes in the LMO that could cause adverse effects, be it direct or indirect, immediate or delayed. It is also relevant if there is a causal link or pathway between the characteristics of the LMO and the potential adverse affect. Points to consider can be for example if there are potential adverse effects on non-target organisms, such as toxicity, allergenicity, any multi-trophic effects, that can affect the survival, development or behavior of these organisms. These points to consider can often be backed up by relevant background material, such as scientific literature.

## The Current Stage of the Roadmap for Risk Assessment of LMOs

The latest version of the current draft roadmap dates back to July 2012. The progress on this “living document,” which is neither meant to be prescriptive nor to impose any obligations on Parties was commended by the sixth Meeting of the Parties to the Protocol in October 2012 in Hyderabad, India. It had also encouraged to use the Guidance, including the roadmap in implementing the provisions on unintentional transboundary movement (Article 17 of the Cartagena Protocol). It also suggested that testing of the draft Guidance should take place. Subsequent to the testing different kinds of feedback was provided by the Parties and observers. Some showed strong support to the document in its current form, others suggested further revision and improvement.

The seventh meeting of the Parties to the Protocol in October 2014 adopted a decision in which it welcomed the results of the testing, invited the Parties and observers to further test or use the draft guidance, including its use as a capacity-building tool. It also suggested a concrete mechanism for revision and improvement of the draft guidance. The links to the background material are constantly being reviewed and updated, as well.

## Reflections on the Way Forward

The development of the draft roadmap has been and continues to be a challenging multi-stakeholder consultative process led by the Parties. This has resulted in a steady and stepwise progress in the development of the document. The recent meeting of the Parties decided on a “two-track” approach, which is a good basis for further progress: on the one hand, the results of the testing will lead to a revision and improvement. On the other hand, there is an ongoing invitation to further test and use the Guidance, also for capacity-building activities. All in all, a practical and efficient mechanism for revision and improvement of the guidance is needed and is currently developed and implemented by the online forum and the expert group. The aim is to provide an improved version of the guidance, including the roadmap, for endorsement and broad support at the next meeting of the Parties in December 2016 in Mexico.

## Conflict of Interest Statement

The author declares that the research was conducted in the absence of any commercial or financial relationships that could be construed as a potential conflict of interest.

## Footnotes

^1^Officially this expert group has been called Ad Hoc Technical Expert Group (AHTEG) on Risk Assessment and Risk Management under the Cartagena Protocol on Biosafety. For reasons of simplifying the language, it will be called “expert group” throughout this article.

^2^The open-ended online forum has been established to complement the work by the expert group. It consists of a few hundred experts on biosafety around the world, nominated by the focal points of the Parties to the Protocol and observers. They work electronically and in a structured form to provide their expert input.
